# To transfer mitochondria or not to transfer mitochondria: ADP does the trick

**DOI:** 10.1371/journal.pbio.3002754

**Published:** 2024-08-15

**Authors:** Jaromir Novak, Jiri Neuzil

**Affiliations:** 1 Institute of Biotechnology, Czech Academy of Sciences, Prague, Czech Republic; 2 School of Pharmacy and Medical Science, Griffith University, Southport, Australia; 3 Faculty of Science and First Faculty of Medicine, Charles University, Prague, Czech Republic

## Abstract

Horizontal mitochondrial transfer (HMT) has emerged as a novel phenomenon in cell biology, but it is unclear how this process of intercellular movement of mitochondria is regulated. A new study in *PLOS Biology* reports that ADP released by stressed cells is a signal that triggers HMT.

Horizontal mitochondrial transfer (HMT) has emerged recently as a new paradigm in cell biology with implications for tissue homeostasis, physiology, and pathophysiology [1]. This novel research field was triggered by a landmark paper that documented movement of “cargo” between cells by means of intercellular conduits referred to as tunneling nanotubes (TNTs) [2]. Then in 2015, HMT was unequivocally documented in vivo, using a mouse tumor model involving syngeneic cancer cell lines with highly compromised respiration, with the primary evidence based on homoplasmic polymorphisms of mtDNA acquired from stromal cells in the tumor microenvironment [3].

Over the last decade, the new discipline of HMT has grown considerably, becoming a focus of an increasing number of laboratories, also offering novel therapeutic potential [1]. A body of work has been dedicated to unraveling the mechanism of HMT and its regulation, with several proposed modes of mitochondrial transfer. They include contact-dependent and contact-independent transfer epitomized by HMT mediated by extracellular vesicles [1]. Within the contact-dependent modes of mitochondrial transfer, tunneling nanotubes as conduits for HMT have been proposed and investigated extensively [1–3]. These intercellular bridges are derived from the plasma membrane and often contain tubulin fibers as “tracks” for movement of “cargo” (including mitochondria) by means of the kinesin/dynein mobility system and several adaptor proteins [1]. While HMT via TNTs is understood to some extent, there is much less information about HMT based on intercellular dendritic structures, another contact-dependent manner of mitochondrial transfer. There are additional modes of HMT, classified as contact independent, mediated by extracellular vehicles [1].

Dendrites as conduits for HMT have been described for intercellular movement of mitochondria from astrocytes to neurons [4] and for the homotypical system presented by mitochondrial movement between osteoclasts, i.e., cells of the same type [5]. A very recent paper has shown that dendrites are also involved in regulation of angiogenesis by osteocyte mitochondria [6], and another recent publication reported that osteocytes suppress bone metastasis by transferring mitochondria via dendrites to cancer cells, where they trigger a STING-dependent anti-cancer immune response [7]. While TNTs present a continuous, barrier-free environment interconnecting cytoplasm of 2 cells, dendrites that start from the mitochondria “donor” cell are not continuous with the acceptor cell, but are attached to its surface by a foot-like structure such that this structure does not fuse with plasma membrane of the acceptor cell (Fig 1).

**Fig 1:**
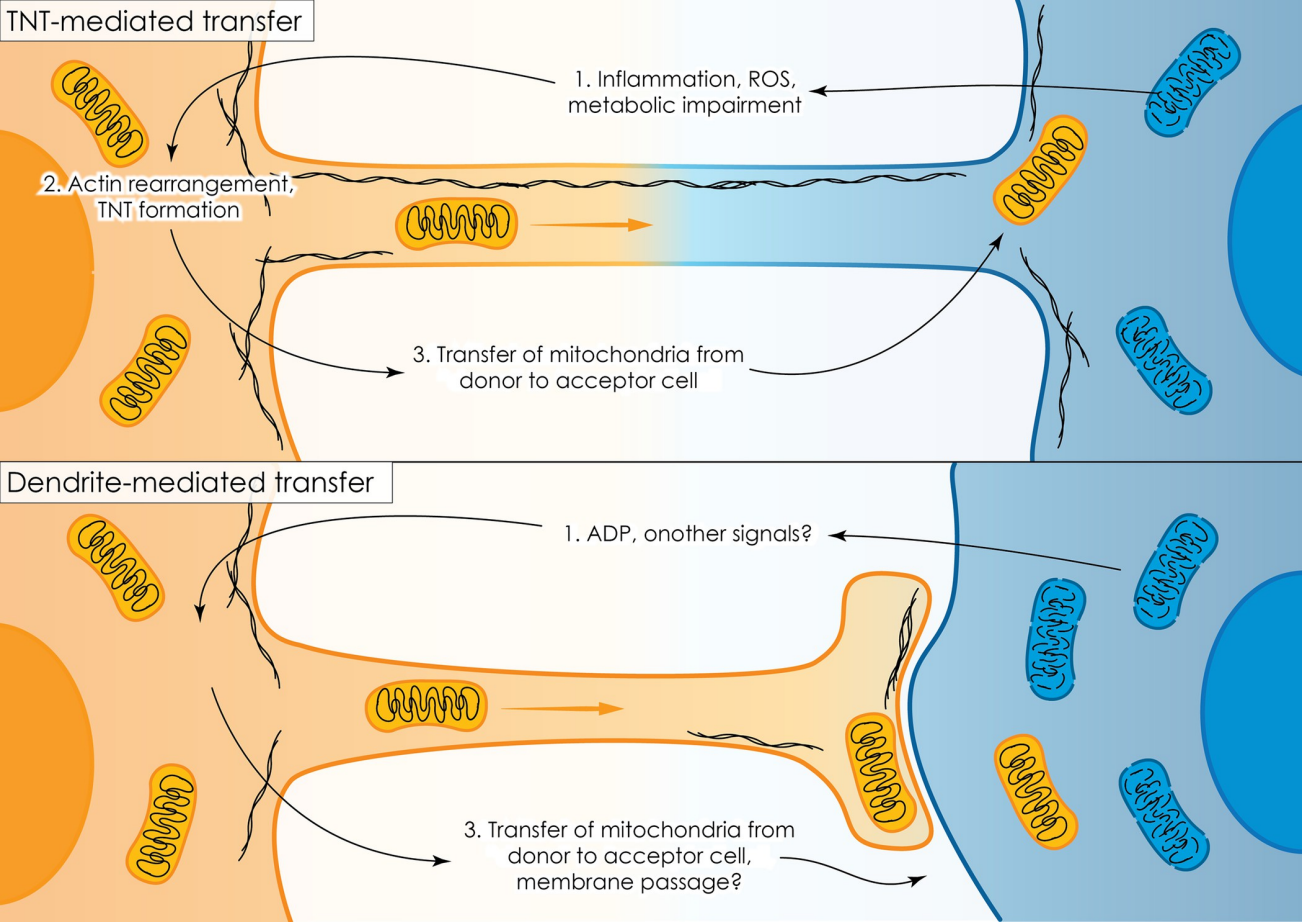
Horizontal transfer of mitochondria via TNTs or dendrites. Among other mechanisms, mitochondria move between cells by membrane protrusions formed by rearrangement of the cytoskeleton in response to stress signals. TNT-mediated transfer (top panel) is known to be induced by various signals, including inflammatory cytokines, reactive oxygen species (ROS) or metabolic stress, resulting in the transport of mitochondria between the TNT-connected cytoplasm of the donor and acceptor cells. Dendrite-mediated transfer (lower panel) has been less studied and its details remain elusive. Li and colleagues [11] demonstrated the role of ADP released from stressed cells as a critical signal triggering dendrite-mediated transfer of mitochondria between osteocytes.

A crucial question that has yet to be answered is what triggers formation of intercellular connections, in other words, how does the process of HMT start. In fact, there is very little known about the onset of intercellular transfer or its regulation. One can hypothesize that the “signal” triggering the process of intercellular mitochondrial transfer emanates from the acceptor cell that is in need of functional mitochondria. The reasons are multiple, but include bioenergetic crisis due to aberrant mitochondrial function, as mentioned earlier for cancer cells devoid of mtDNA [1,3], or damage of neuronal cell mitochondria after stroke [4]. There is thus very little literature describing this process, which is essential for the onset and execution of HMT. One report shows that onset of formation of tubular structures, which can be utilized for intercellular movement of mitochondria, is regulated by the M-sec protein [8]. This polypeptide interacts with the Ral protein and the exocyst complex on the surface of the “donor” cell, causing local re-arrangement of plasma membrane proteins that allows for formation and extension of cellular protrusions. Yet, once again, the trigger for the very onset of this process, most likely coming from the “acceptor” cells in the form of a signal, such as a small “stress”/linked molecule [9], is unknown. Therefore, a paper just published in *PLOS Biology* provides important new data indicating the involvement of nucleotide signaling [10] in this process [11].

Li and colleagues [11] studied a system in which they observed transfer of mitochondria to stressed osteocytes via dendritic structures, apparently to correct the “bioenergetic crisis” in these cells in order to promote tissue homeostasis. In this paper, the authors show that osteocytes respond to stress release signal in the form of ADP, which is considered a trigger for mitochondrial transfer, via dendrites in this case. The target cell that “donates” mitochondria to the stressed cell recognizes the incoming ADP molecules via the purinergic P2Y2 and P2Y6 receptors in its plasma membrane. The stressed osteocytes, undergoing bioenergetic crisis, in this way remedy their aberrant mitochondrial function, resulting in normalization of mitochondrial respiration, and this allows these cells to restore their physiological state and function. Interestingly, the authors show that activation of the purinergic receptors by ADP coming from stressed cell is required for subsequent mitochondrial transfer, as deletion of these receptors prevented intercellular movement of mitochondria. Further, signaling pathways triggered by activation of the purinergic receptors result in interaction of mitochondria and the endoplasmic reticulum, which is a prerequisite for dendrite formation and for the ensuing HMT.

While a number of questions still remain unanswered, for example, how the dendrite structures extend and exactly how mitochondria cross the barrier on the side of the acceptor cell, presented by the foot of the dendrite and the adjacent plasma membrane, this is one of very few papers that have successfully attempted to explain the nature of the so called “danger” signals sent out by a stressed cell in need of improving its aberrant mitochondrial function, followed by a cascade of (signaling) events culminating in formation of a conduit for mitochondrial transfer and delivery of the organelles to the cell in need. In the case of stressed osteocytes, these danger signals are presented by ADP, as is unequivocally shown [10]. However, whether this is also the case for other systems where HMT occurs (and there are numerous examples of various tissues executing both heterotypical or homotypical contact-dependent mitochondrial transfer; reviewed in reference [2] and their nature, or whether there are other signals (which can be expected), has yet to be established. With this in mind, this *PLOS Biology* paper will stimulate much needed research in this area, which is intriguing from point of view of fundamental cell biology, and which is hoped to be harnessed for alleviating various pathological states.
